# *Bifidobacterium infantis* as a probiotic in preterm infants: a systematic review and meta-analysis

**DOI:** 10.1038/s41390-023-02716-w

**Published:** 2023-07-17

**Authors:** Vamsi K. Batta, Shripada C. Rao, Sanjay K. Patole

**Affiliations:** 1 Neonatal Intensive Care Unit, Perth Children’s Hospital, Perth, WA Australia; 2 Neonatal Intensive Care Unit, King Edward Memorial Hospital, Perth, WA Australia; 3 School of Medicine, University of Western Australia, Perth, WA Australia

## Abstract

**Background:**

*Bifidobacterium infantis* has special abilities to utilise human milk oligosaccharides. Hence we hypothesised that probiotic supplements containing *B. infantis* may confer greater benefits to preterm infants than probiotic supplements without *B. infantis.*

**Methods:**

A systematic review with meta-analysis was conducted according to standard guidelines. We selected RCTs evaluating probiotics compared to placebo or no treatment in preterm and/or low birth weight infants. Probiotic effects on Necrotizing Enterocolitis (NEC), Late Onset Sepsis (LOS) and Mortality were analysed separately for RCTs in which the supplemented probiotic product contained *B. infantis* and those that did not contain *B. infantis*.

**Results:**

67 RCTs were included (*n* = 14,606), of which 16 used probiotics containing *B. infantis* (Subgroup A) and 51 RCTs did not (Subgroup B) Meta-analysis of all RCTs indicated that probiotics reduced the risk of NEC, LOS, and mortality. The subgroup meta-analysis demonstrated greater reduction in the incidence of NEC in subgroup A than subgroup B [(relative risk in subgroup A: 0.38; 95% CI, 0.27–0.55) versus (0.67; 95% CI, 0.55–0.81) in subgroup B; *p* value for subgroup difference: 0.01].

**Conclusions:**

These results provide indirect evidence that probiotic supplements that include *B. infantis* may be more beneficial for preterm infants. Well-designed RCTs are necessary to confirm these findings.

**Impact:**

- Evidence is emerging that beneficial effects of probiotics are species and strain specific.
- This systematic review analyses if *B. infantis* supplementation provides an advantage to preterm infants.
- This is the first systematic review evaluating the effects of probiotics containing *B*. *infantis* in preterm infants.
- The results of this systematic review provides indirect evidence that probiotics that include *B. infantis* may be more beneficial for preterm infants. These results will help in guiding future research and clinical practice for using *B. infantis* as a probiotic in preterm infants.

## Introduction

Preterm infants are at risk of mortality, and morbidities such as necrotising enterocolitis (NEC) and late-onset sepsis (LOS). An important risk factor for NEC and sepsis in preterm infants is gut dysbiosis.^1^ Hence attenuating dysbiosis by the use of probiotics has the potential to improve their clinical outcomes.

Probiotics are live microorganisms that when administered in adequate amounts, could confer beneficial effects on the host.^2^ Systematic reviews of randomised controlled trials (RCTs) and non-randomised studies have shown that probiotic supplementation reduces the risk of NEC (≥Stage II), LOS, and mortality in preterm infants.^3^ The benefits of probiotics relate to their ability to improve the gut barrier, modulate the immune system and attenuate gut dysbiosis.^1^ Probiotics have been shown to reduce the relative abundance of pathogens in the gut through various pathways, including blocking the receptors and competing for nutrients.^4^

Considering that probiotic effects are considered species and strain specific,^5^ data on individual probiotic species and strains is important for guiding clinical practice and research.

During the early human development, bacteria belonging to the genus Bifidobacterium play an important role.^6^ Among the bifidobacteria, *Bifidobacterium longum* subspecies *infantis* (*B. infantis*) is considered as an important gut symbiont, especially in infancy. It is considered as a champion coloniser of the gut due to its properties for the consumption of human milk oligosaccharides (HMOs).^7^ It may have a competitive advantage against other bacteria, allowing increased colonisation and resulting in fewer luminal pathogens.^8^ *B. infantis* promotes maturation of the innate immune response^9^ and improves the anti-inflammatory properties through the production of tryptophan metabolite, indole-3-lactic acid (ILA).^10^ Given these properties, we hypothesised that supplementation with probiotics containing *B. infantis* will be more beneficial in preterm infants than those without this sub-species of bacteria.

To our knowledge, no systematic review, including the latest systematic review and the network meta-analysis^3^ has addressed this specific question. There are no RCTs in preterm infants that have compared B. *infantis* versus placebo. In addition, apart from the small RCT by our group,^11^ there are no RCTs that have compared supplementation with probiotics containing *B. infantis versus* probiotics not containing *B. infantis*.

Hence, we conducted a systematic review that had two subgroups: Subgroup A: Probiotics containing *B*. *infantis* versus placebo/no probiotics; Subgroup B: Probiotics not containing *B. infantis* versus placebo/no probiotics.

## Methods

Guidelines from the Cochrane Neonatal Review Group,^12^ and the PRISMA (Preferred Reporting Items for Systematic Reviews and Meta-Analyses) statement^13^ were followed for undertaking and reporting this systematic review and meta-analysis.

### Eligibility criteria

#### Types of studies

We selected RCTs evaluating probiotics for the prevention of morbidity or mortality in preterm (gestational age <37 weeks) and/or low birth weight (birth weight <2500 g) infants. We excluded studies that enrolled term infants. Non-randomised studies, narrative reviews, systematic reviews, case reports, letters, editorials, and commentaries were excluded but read to identify potentially eligible studies.

#### Types of participants

Preterm infants born before gestation <37 weeks, low birth weight (<2500 g), or both.

#### Interventions and comparisons

We included studies assessing enteral administration of any probiotic commenced within the first week of life and continued for at least one week compared to placebo or no treatment. We excluded studies that used prebiotics or synbiotics (i.e. combination of a prebiotic with probiotics).

#### Outcomes

These included (1) NEC ≥Stage II (Modified Bell’s criteria)^14^; (2) LOS defined as isolation of a pathogen from blood, cerebrospinal fluid, or a normally sterile body space after 48 h of birth; (3) All-cause mortality.

#### Search strategy

The Cochrane Central Register of Controlled clinical trials (www.thecochranelibrary.com, through December 2022), PubMed (https://www.ncbi.nlm.nih.gov, 1966–December 2022), EMBASE (Excerpta Medica dataBASE) via Ovid (http://ovidsp.tx.ovid.com, 1980–December2022), EMCARE via OVID (http://ovidsp.tx.ovid.com, 1980–December 2022) databases were searched. We searched https://clinicaltrials.gov and ANZCTR (Australia New Zealand Clinical Trials Registry (www.anzctr.org.au) for ongoing RCTs. Grey literature was searched using Mednar (www.mednar.com). The reference lists of identified studies and key review articles were searched to identify additional RCTs. No language restriction was applied.

PubMed was searched using the following keywords:

((((((((Probiotic) OR (Probiotics)) OR (Bifidobacteria)) OR (Bifidobacterium)) OR (Lactobacilli)) OR (Lactobacillus)) OR (Saccharomyces)) AND ((((((Preterm infant) OR (Preterm infants)) OR (premature infants)) OR (low birth weight infants)) OR (very low birth weight infants)) OR (extremely low birth weight infants))) AND (Trial). PubMed was also searched using relevant MeSH words. Other databases were searched using similar terminologies.

### Study selection

Abstracts of the citations obtained from the initial broad search were read independently by two reviewers to identify potentially eligible studies. Full-text articles of these studies were obtained and assessed independently for eligibility by two reviewers, using the predefined eligibility criteria. Differences in opinion were resolved by a group discussion to reach a consensus. Multiple publications of the same study were excluded to avoid duplication of the data.

### Data extraction

Two reviewers independently extracted the data using a standardised data collection form. Discrepancies were resolved by discussion and consensus among all authors.

### Assessment of risk of bias (ROB) of RCTs

ROB was assessed using the Cochrane “Risk of Bias Assessment Tool”.^12^ Two reviewers independently assessed the ROB in all domains including random number generation, allocation concealment, blinding of intervention and outcome assessors, completeness of follow up and selectivity of reporting. For each domain, the ROB was assessed as low, high or unclear.

### Data synthesis and statistical analysis

Meta-analysis was performed using statistical software, STATA (Version 17.0). Since heterogeneity was expected we used random-effects model (REM) model for meta-analysis. Fixed effect model (FEM) was also used to assure that the choice of the model did not influence the results. Since all outcomes of interest were binary, we used relative risk (RR) and 95% CI to summarise their results.

### Heterogeneity

Clinical heterogeneity was assessed and reported by summarising characteristics such as the study population, dose, and duration of probiotic supplementation. Statistical heterogeneity was estimated using the *I*^2^ statistic and interpreted as per Cochrane handbook^11^ as follows: 0–40%: might not be important; 30–60%: may represent moderate heterogeneity; 50–90%: may represent substantial heterogeneity; 75–100%: considerable heterogeneity.

### Publication bias

To assess for any publication bias, we used Egger’s,^15^ Harbord’s,^16^ Begg’s^17^ and trim & fill plots.^18^

### Summary of Findings (SOF) table

The key information about the quality of evidence, the magnitude of the effect of the intervention and the sum of available data on the main outcome was presented in the SOF table according to the Grades of Recommendation, Assessment, Development and Evaluation (GRADE) guidelines.^19^

## Results

Initial broad search identified 2315 records, of which full texts of 84 potentially eligible studies were read in detail. Seventeen of these studies were excluded being non-RCTs (*n* = 4), RCTs of prebiotics or synbiotics (*n* = 6), being conducted in full-term infants (*n* = 1), and not reporting our outcomes of interest (*n* = 6). Finally, 67 RCTs were included.^20–86^

**Fig. 1:**
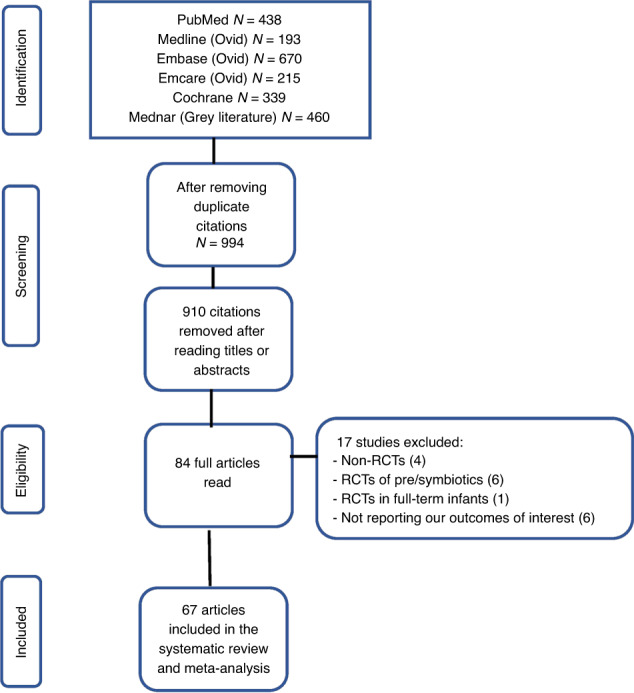
PRISMA flow diagram of study selection. Identification of studies via databases and registers.

The flow diagram of the study selection process is given in Fig. 1.

Of the 67 RCTs (*n* = 14,606), 16 (*n* = 4962) had used *B. infantis* as a component of the probiotic supplement.^20–35^ The remaining 51 (*n* = 9644) did not use *B. infantis*.^36–86^ The mean gestation and birth weight ranged from 25.4 weeks to 33.5 weeks and from 727 g to 2262 g, respectively. The duration of probiotic supplementation varied from a minimum of 2 weeks to until discharge/40 weeks corrected gestational age. The probiotic dose ranged from 18 × 10^6^ (18 million) colony forming units (CFU) to 12 × 10^9^ (12 billion) CFU/day. NEC, LOS and all-cause mortality were included as the outcomes in 63, 57 and 54 studies, respectively. The characteristics of the included studies are given in Table 1a, b.

**Table 1:** (a) Randomised controlled trials using probiotics containing *B. infantis*; (b) randomised controlled trials using probiotics without *B. infantis*.

(a)												
S. no	Study ID (ref. no.)	Mean birth gestation (weeks)	Mean birth weight (g)	Type of probiotic	Total dose/day (CFU)	Total dose of *B. infantis*/day (CFU)	Probiotic formulation	Probiotic arm (*n*)	Duration of intervention (weeks)	Controls	Control arm (*n*)	Outcomes/results (probiotic vs control) %
1	Al-Hosani^20^	25.7	778.5	MSP	1 × 10^9^	5 × 10^8^	*L. rhamnosus* *B. longum* subsp*. infantis*	50	Upto 34 weeks CGA or discharge	No treatment	51	NEC: 4.0 vs 3.9 LOS: 26 vs 31.3 Mortality: 6 vs 7.8
2.	Alshaik^21^	25.8	763	MSP	4 × 10^9^	6 × 10^8^	*B. breve* *B. bifidum* *B. longum* subsp.infantis *B. longum* subsp.longum *L. rhamnosus*	31	Upto 37 weeks CGA or discharge	No treatment	31	NEC: 6.5 vs 0.0 LOS: 25.8 vs 9.7 Mortality: 6.5 vs 0.0
3	Bin-Nun^22^	29.5	1131.4	MSP	1.05 × 10^9^	0.35 × 10^9^	*B. longum* subsp*. infantis;* *B. bifidum* *S. salivarius* subsp*. thermophilus*	72	Upto 36 weeks CGA	Placebo	73	NEC: 1.4 vs 13.6 LOS: 43 vs 32.9 Mortality: 4.2 vs 10.9
4	Chowdhury^23^	31.5	1324	MSP	TBA	TBA	*L. rhamnosus;* *L. acidophilus; L. casei* *B. longum* subsp*. infantis; B. bifidum;* *B. longum* subsp. *longum*	60	Until discharge	No treatment	59	NEC: 1.7 vs 10.2 Mortality: 8.3 vs 11.9
5	Dutta^24^	30.9	1323.3	MSP	10 × 10^9^	NA	*B. infantis*, *L. rhamnosus L. casei, L. plantarum* *L. acidophilus*, *S. boulardii*	114	4	Placebo	35	NEC: 5.3 vs 0.0 LOS: 8.8 vs 17.1 Mortality: 5.5 vs 5.7
6	Fernandez-Carrocera^25^	31.1	1130	MSP	2.6 × 10^9^	2.76 × 10^7^	*L. rhamnosus;* *L. acidophilus;* *L. casei;* *L. plantarum* *B. longum* subsp*. infantis* *S. salivarius subsp. thermophilus*	75	Until discharge	No treatment	75	NEC: 8 vs 16 LOS: 56 vs 58.7 Mortality: 1.3 vs 9.3
7	Jacobs^26^	27.9	1055.5	MSP	1 × 10^9^	3 × 10^8^	*B. infantis, B. lactis* *S. thermophilus*	548	Term corrected age/until discharge	Placebo	551	NEC: 2 vs 4.3 LOS: 14.2 vs 16.5 Mortality: 4.9 vs 5.0
8	Kanic^27^	28.5	1064.2	MSP	1.2 × 10^7^	0.45 × 10^7^	*L. gasseri* *B. infantis* *E. faecium*	40	Until discharge	No treatment	40	NEC: 0 vs 12.5 LOS: 50 vs 80 Mortality: 5.0 vs 7.5
9	Lin^28^	28.3	1087.2	MSP	NA	NA	*L. acidophilus* *B. longum* subsp*. infantis*	180	Until discharge	No treatment	187	NEC: 1.1 vs 5.3 LOS: 12.2 vs 19.2 Mortality: 3.9 vs 10.7
10	Ren^29^	31	1700	MSP	NA	NA	*B. longum* subsp*. infantis* *L. acidophilus* *Bacillus cereus* *E. faecalis*	80	1–2 weeks	No treatment	70	NEC: 3.7 vs 7.1 LOS: 2.5 vs 12.8
11	Samanta^30^	30.1	1191.4	MSP	2 × 10^9^	0.5 × 10^9^	*L. acidophilus* *B. infantis;* *B. lactis;* *B. longum*	91	Until discharge	MBM	95	NEC: 5.5 vs 15.8 LOS: 14.3 vs 29.5 Mortality: 4.4 vs 14.7
12	Sinha^31^	NA	2262	MSP	1 × 10^9^	NA	*L. acidophilus;* *L. plantarum; L. casei;* *B. breve; B. infantis;* *B. longum, S. thermophilus*	668	4	Placebo	672	– LOS: 5.7 vs 8.0 Mortality: 0.1 vs 0.3
13	Sowden^32^	29	1174	MSP	2 × 10^9^	0.67 × 10^8^	*L. acidophilus* *B. infantis* *B. bifidum*	100	4 or until discharge	Placebo	100	NEC: 0 vs 2 Mortality: 0 vs 1
14	Van Neikerk^33^	28.7	1009	MSP	1 × 10^9^	0.5 × 10^9^	*L. rhamnosus* *B. infantis*	91	4	Placebo	89	NEC: 0 vs 4.3 LOS: 17.6 vs 11.8 Mortality: 5.5 vs 6.5
15	Van Neikerk^34^	28.7	972	MSP	1 × 10^9^	0.5 × 10^9^	*L. rhamnosus* *B. infantis*	54	4	Placebo	56	NEC: 0 vs 3.6 LOS: 20.3 vs 14.3 Mortality: 7.4 vs 12.5
16	Xiao-yuan^35^	31	1745	MSP	NA	NA	*L. acidophilus* *B. infantis* *E. faecalis*	276	Until discharge	No treatment	248	NEC: 1.8 vs 6.8

(b)											
S. no.	Study ID (ref. no.)	Birth gestation (mean) (weeks)	Birth weight (mean) (g)	Type of probiotic	Total dose/day (CFU)	Probiotic formulation	Probiotic intervention (*n*)	Duration of intervention	Control	Control arm (*n*)	Outcomes/results (probiotics vs control) %
1	Arora^36^	32.9	1700	MSP	2.5 × 10^8^	*L. rhamnosus;* *L. acidophilus* *B. longum* subsp*. longum* *S. boulardii*	75	2	No treatment	75	NEC: 0 vs 5.3 LOS: 2.7 vs 29.3 Mortality: 0 vs 2.7
2	Braga^37^	29.4	1173.7	MSP	3.5 × 10^9^	*L. casei* *B. breve*	119	4	No treatment	112	NEC: 0 vs 3.6 LOS: 33.6 vs 37.5 Mortality: 21.8 vs 24.1
3	Chandrashekar^38^	NA	NA	MSP	NA	*L. acidophilus*, *L. rhamnosus*, *B. longum*, *S. boulardii*	70	Until CGA of 35weeks	No treatment	70	NEC: 0 vs 4.3 Mortality: 1.4 vs 5.7
4	Chrzanowska-Liszewska^39^	29.5	1257.8	SSP	6 × 10^9^	*L. rhamnosus*	21	6 weeks	Preterm formula	26	NEC: 0 vs 0 LOS: 9.5 vs 11.5
5	Costalos^40^	31.4	1648.1	SSP	NA	*S. boulardii*	51	≥4	Preterm formula	36	NEC: 9.8 vs 16.7 LOS: 5.9 vs 8.3
6	Costeloe^41^	28	1041	SSP	NA	*B. breve*	650	Upto 36 weeks CGA or discharge	Placebo	660	NEC: 9.4 vs 10 LOS: 11.2 vs 11.7 Mortality: 8.3 vs 8.5
7	Cui^42^	32.7	1698	SSP	1 × 10^8^	*L. reuteri*	45	Until discharge	No treatment	48	NEC: 2.2 vs 10.4 LOS: 4.4 vs 8.3
8	Dani^43^	30.8	1334.9	SSP	6 × 10^9^	*L. rhamnosus*	295	Until discharge	Placebo	290	NEC: 1.4 vs 2.8 LOS: 4.7 vs 4.1 Mortality: 0 vs 0.7
9	Dashti^44^	31.2	1406.4	MSP	0.5–1 × 10^9^	*L. acidophilus;* *L. rhamnosus;* *L. bulgaricus;* *L. casei* *B. breve;* *B. longum*, *S. thermophilus*	69	NR	Placebo	67	NEC: 2.9 vs 1.5 Mortality: 11.6 vs 6.0
10	Demirel^45^	29.3	1147.4	SSP	5 × 10^7^	*S. boulardii*	135	Until discharge	No treatment	136	NEC: 4.4 vs 5.1 LOS: 14.8 vs 15.4 Mortality: 3.7 vs 3.7
11	Deng^46^	32.8	1628.7	MSP	NA	*L. acidophilus* *B. longum* subsp*. longum* *E. faecalis*	63	2	No treatment	62	NEC: 1.6 vs 12.9
12	Dilli^47^	28.7	1204.3	SSP	NA	*B. animalis* subsp*. lactis*	100	8	Placebo	100	NEC: 2 vs 18 LOS: 8 vs 13 Mortality: 3 vs 12
13	Fujii^48^	31.3	1427.7	SSP	2 × 10^9^	*B. breve*	11	Until discharge	Placebo	8	NEC: 0 vs 0 LOS: 9 vs 12.5
14	Hariharan^49^	29	959.2	MSP	5 × 10^9^	*L. acidophilus* *B. bifidum* *S. boulardii*	93	6	No treatment	103	NEC: 3.2 vs 6.1 LOS: 9.7 vs 15.5 Mortality: 4.3 vs 4.8
15	Hays^50^	29.2	1170	MSP	1 × 10^9^	*B. animalis* subsp*. lactis;* *B. longum* subsp*. longum*	145	4–6	Placebo	52	NEC: 5.5 vs 5.8 LOS: 11.7 vs 36.5 Mortality: 2.0 vs 1.9
16	Hernandaz-Enriquez^51^	31.4	1293.3	SSP	0.6–1 × 10^8^	*L. reuteri*	24	3	No treatment	20	NEC: 4.2 vs 25 LOS: 87.5 vs 95 Mortality: 8.3 vs 0
17	Hikaru^52^	28.3	1036.4	SSP	1 × 10^9^	*B. breve*	108	Until discharge	Placebo	100	NEC: 0 vs 0 LOS : 9.2 vs 22 Mortality: 0 vs 4
18	Hua^53^	33.1	1786.6	MSP	NA	*L. delbrueckii* subsp*. bulgaricus* *B. longum* subsp*. longum* *S. salivarius* subsp*. thermophilus*	119	2	No treatment	138	NEC: 0 vs 1.4 LOS: 1.7 vs 5.8 Mortality: 1.7 vs 2.1
19	Huang^54^	30.1	1100	SSP	NA	*B. adolescentis*	95	1–2	No treatment	88	NEC: 0 vs 3.4
20	Kaban^55^	33	1562.5	SSP	1 × 10^8^	*L. reuteri*	47	Until discharge	Placebo	47	NEC: 0 vs 6.4 LOS: 2.1 vs 6.4 Mortality: 2.1 vs 8.5
21	Kitajima^56^	28.2	1026	SSP	0.5 × 10^9^	*B. breve*	45	4	No treatment	46	NEC: 0 vs 0 LOS: 2.2 vs 0 Mortality: 0 vs 4.3
22	Lin^57^	NA	1053.1	MSP	2 × 10^9^	*L. acidophilus* *B. bifidum*	217	6	Placebo	217	NEC: 1.8 vs 6.4 LOS: 18.4 vs 11.0 Mortality: 0.9 vs 4.1
23	Manzoni^58^	29.4	1193	SSP	6 × 10^9^	*L. rhamnosus*	39	6 or until discharge	No treatment	41	NEC: 2.6 vs 4.9 LOS: 48.7 vs 53.6 Mortality: 12.8 vs 14.6
24	Marti^59^	25.5	727	SSP	1.25 × 10^8^	*L. reuteri*	54	Until 36 weeks corrected age	Placebo	54	LOS: 46.3 vs 42.6
25	Mihatsch^60^	26.7	863.4	SSP	12 × 10^9^	*B. animalis* subsp*. lactis*	91	Until discharge	Placebo	89	NEC: 2.2 vs 4.5 LOS: 30.8 vs 32.6 Mortality: 2.2 vs 1.1
26	Millar^61^	30.3	1472.5	SSP	2 × 10^8^	*L. rhamnosus*	10	2	Placebo	10	NEC: 0 vs 0 LOS: 0 vs 0
27	Mohan^62^	31.2	1425.3	SSP	1.6–4.8 × 10^9^	*B. animalis* subsp*. Lactis*	37	3	Placebo	32	NEC: 5.4 vs 3.1
28	Oncel^63^	28.1	1059.5	SSP	1 × 10^8^	*L. reuteri*	200	4	Placebo	200	NEC: 4 vs 5 LOS: 6.5 vs 12.5 Mortality: 7.5 vs 10
29	Oshiro^64^	28.1	1025.5	SSP	2.5 × 10^8^	*B. breve*	17	8	Placebo	18	NEC: 0 vs 0
30	Patole^65^	28.5	1060.2	SSP	3 × 10^9^	*B. breve*	77	37 weeks corrected age	Placebo	76	NEC: 0 vs 1.3 LOS: 30 vs 18.1 Mortality: 0 vs 0
31	Qiao^66^	32.3	1623	MSP	NA	*L. acidophilus* *B. longum* subsp*. longum* *E. faecium*	149	2	Placebo	138	LOS: 6.7 vs 15.2 Mortality: 4.0 vs 6.5
32	Rehman^67^	32.6	1320	MSP	NA	*Bifidobacterium* *L. acidophilus*, *S. thermophillus* *L. delbrueckii*	73	Until discharge	No treatment	73	NEC: 2.7 vs 11 Mortality: 5.5 vs 8.2
33	Reuman^68^	30.6	1371.5	SSP	18 × 10^6^	*L. acidophilus*	15	4	Placebo	15	NEC: 0 vs 0 LOS: 6.7 vs 20 Mortality: 6.7 vs 20
34	Rojas^69^	32	1522.9	SSP	1 × 10^8^	*L. reuteri*	372	Until discharge	Placebo	378	NEC: 2.4 vs 4.0 LOS: 6.4 vs 4.5 Mortality: 5.9 vs 7.4
35	Romeo^70^	33.5	1961.7	SSP	6 × 10^9^	*L. reuteri*	166	6 or until discharge	No treatment	83	LOS: 1.8 vs 10.8
36	Rouge^71^	28.1	1084.8	MSP	4 × 10^8^	*L. rhamnosus* *B. longum*	45	Until discharge	Placebo	49	NEC: 4.4 vs 2.0 LOS: 33.3 vs 26.5 Mortality: 4.4 vs 8.2
37	Roy^72^	32.1	1130.5	MSP	1.5–3.0 × 10^9^	*L. acidophilus* *B. bifidum;* *B. animalis* subsp*. lactis;* *B. longum* subsp*. longum*	56	6 or until discharge	Placebo	56	NEC: 3.6 vs 3.6 LOS: 55.3 vs 75 Mortality: 12.5 vs 14.3
38	Sadowska-Krawczenko^73^	29.5	973.1	SSP	NA	*L. rhamnosus*	30	Until discharge	Placebo	25	NEC: 3.3 vs 16 LOS: 30 vs 28 Mortality: 3.3 vs 0
39	Saengtawesin^74^	30.8	1229.6	MSP	2 × 10^9^	*L. acidophilus* *B. bifidum*	31	6 or until discharge	No treatment	29	NEC: 3.2 vs 3.4 LOS: 6.4 vs 3.4 Mortality: 0 vs 0
40	Sari^75^	29.6	1254.5	SSP	3.5 × 10^8^	*L. sporogenes*	110	Until discharge	Placebo	111	NEC: 5.5 vs 9.0 LOS: 26.3 vs 23.4 Mortality: 2.7 vs 2.7
41	Serce^76^	28.8	1144	SSP	1 × 10^9^	*S. boulardii*	104	Until discharge	Placebo	104	NEC: 6.7 vs 6.7 LOS: 18.3 vs 24 Mortality: 4.8 vs 3.8
42	Shadkam^77^	30.9	1407.5	SSP	5.6 × 10^7^	*L. reuteri*	30	Until full enteral feeding	Placebo	30	NEC: 6.7 vs 36.7 LOS: 13.3 vs 33.3 Mortality: 3.3 vs 6.7
43	Shashidar^78^	31.1	1223	MSP	1.25 × 10^9^	*L. acidophilus;* *L. rhamnosus* *B. longum* *S. boulardii*	49	4	Placebo	49	NEC: 4.0 vs 12.2 LOS: 12.2 vs 14.3 Mortality: 2.0 vs 6.1
44	Singh^79^	NA	NA	SSP	NA	*L. rhamnosus*	37	Until full enteral feeds	Placebo	35	NEC: 16.2 vs 28.6 Mortality: 8.1 vs 8.6
45	Spreckels^80^	25.4	728	SSP	1.25 × 10^8^	*L. reuteri*	64	Until 36 weeks corrected age	Placebo	63	NEC: 4.0 vs 9.0 LOS: 35.0 vs 30.0 Mortality: 6.0 vs 4.0
46	Stratiki^81^	30.8	1500	SSP	2 × 10^7^	*B. lactis*	41	Until discharge	Preterm formula	36	NEC: 0 vs 8.3 LOS: 0 vs 8.3 Mortality: 0 vs 8.3
47	Strus^82^	29.7	1350.1	MSP	2 × 10^6^	*L. rhamnosus*, *B. breve*	80	6 or until discharge	Placebo	73	NEC: 2.5 vs 1.4 LOS: 13.7 vs 9.6 Mortality: 2.5 vs 5.5
48	Tewari^83^	30	1363	SSP	2.4 × 10^9^	*Bacillus clausii*	123	6	Placebo	62	NEC: 0 vs 0 LOS: 6.5 vs 4.8 Mortality: 9.7 vs 11.6
49	Totsu^84^	28.6	1007.7	SSP	2.5 × 10^9^	*B. bifidum*	153	Until 2000 g weight	Placebo	130	NEC: 0 vs 0 LOS: 3.9 vs 10 Mortality: 1.7 vs 0
50	Wejryd^85^	25.5	733	SSP	1.25 × 10^8^	*L. reuteri*	68	4	Placebo	66	NEC: 10.3 vs 12.1 LOS: 36.8 vs 34.8 Mortality: 7.3 vs 7.6
51	Xu^86^	33	1951.9	SSP	1.95 × 10^9^	*S. boulardii*	51	4 or until discharge	No treatment	49	NEC: 0 vs 0 LOS: 7.8 vs 12.2

### ROB of included studies

Of the 16 studies that used probiotics containing *B. infantis*, 9 (56.2%) studies were considered to have “low ROB” on the domain of random sequence generation, 5 (31.2%) had “high ROB” for performance & detection bias. Fifteen (93.7%) studies and 12 (75.0%) showed “low ROB” for attrition and reporting bias, respectively. Among the 51 studies that used probiotics without *B. infantis*, 36 (70.5%) and 28 (54.9%) were considered “low ROB” in the selection bias category. 24 (47%) studies had “unclear risk” or “high risk” ROB for blinding. For the attrition and reporting bias categories, 44 (86.2%) and 35 (68.6%) studies showed “low ROB”. Details of the ROB analysis are given in Table 2a, b.

**Table 2:** (a) Risk of bias in studies using probiotics containing *B. infantis*; (b) risk of bias in studies using probiotics without *B. infantis*.

(a)						
S. no.	Study ID	Random sequence generation (selection bias)	Allocation concealment (selection bias)	Blinding (performance and detection bias) all outcomes	Incomplete outcome data (attrition bias) all outcomes	Selective reporting (reporting bias)
1	Al-Hosni^20^	Unclear risk	Unclear risk	Unclear risk	Low risk	Low risk
2	Alshaik^21^	Low risk	Low risk	Unclear risk	Low risk	Low risk
3	Bin-Nun^22^	Unclear risk	Unclear risk	Unclear risk	Low risk	Unclear risk
4	Chowdhury^23^	High risk	High risk	High risk	Low risk	Low risk
5	Dutta^24^	Low risk	Unclear risk	Low risk	Low risk	Low risk
6	Fernandez-Carrocera^25^	Low risk	Low risk	Unclear risk	Low risk	Low risk
7	Jacobs^26^	Low risk	Low risk	Low risk	Low risk	Low risk
8	Kanic^27^	High risk	High risk	High risk	Low risk	Low risk
9	Lin^28^	Low risk	Low risk	Unclear risk	Low risk	Low risk
10	Ren^29^	Low risk	Unclear risk	High risk	Unclear risk	Unclear risk
11	Samanta^30^	Unclear risk	Unclear risk	High risk	Low risk	Low risk
12	Sinha^31^	Low risk	Low risk	Low risk	Low risk	Low risk
13	Sowden^32^	Unclear risk	Low risk	Low risk	Low risk	Unclear risk
14	Van Neikerk^33^	Low risk	Low risk	Low risk	Low risk	Low risk
15	Van Neikerk^34^	Low risk	Low risk	Low risk	Low risk	Low risk
16	Xiao-yuan^35^	High risk	High risk	High risk	Low risk	High risk

(b)						
S. no.	Study ID	Random sequence generation (selection bias)	Allocation concealment (selection bias)	Blinding (performance and detection bias) all outcomes	Incomplete outcome data (attrition bias) all outcomes	Selective reporting (reporting bias)
1.	Arora^36^	Low risk	High risk	High risk	Low risk	Low risk
2.	Braga^37^	Low risk	Low risk	Unclear risk	Low risk	Low risk
3.	Chandrashekar^38^	High risk	Unclear risk	High risk	Low risk	Low risk
4.	Chrzanowska-Liszewska^39^	Low risk	Low risk	Low risk	Low risk	Low risk
5.	Costalos^40^	Unclear risk	Low risk	Low risk	Low risk	Low risk
6.	Costeloe^41^	Low risk	Low risk	Low risk	Low risk	Low risk
7.	Cui^42^	Low risk	Unclear risk	Low risk	Low risk	Unclear risk
8.	Dani^43^	Unclear risk	Low risk	Low risk	Low risk	Low risk
9.	Dashti^44^	Unclear risk	Unclear risk	Low risk	Unclear risk	Unclear risk
10.	Demirel^45^	Low risk	Low risk	High risk	Low risk	Low risk
11.	Deng^46^	High risk	High risk	High risk	Low risk	High risk
12.	Dilli^47^	Low risk	Low risk	Low risk	Low risk	Low risk
13.	Singh^79^	Low risk	Low risk	Low risk	Low risk	Low risk
14.	Fujii^48^	Unclear risk	Unclear risk	High risk	Unclear risk	Unclear risk
15.	Hariharan^49^	Unclear risk	Unclear risk	High risk	Low risk	Unclear risk
16.	Hays^50^	Low risk	Low risk	Low risk	Low risk	Low risk
17.	Hernandaz-Enriqez^51^	Unclear risk	Low risk	High risk	Unclear risk	Unclear risk
18.	Hikaru^52^	Unclear risk	Unclear risk	High risk	Low risk	Unclear risk
19.	Hua^53^	Low risk	High risk	Low risk	Low risk	Unclear risk
20.	Huang^54^	Unclear risk	Unclear risk	High risk	Unclear risk	Unclear risk
21.	Shashidar^78^	Low risk	Low risk	Low risk	Unclear risk	Low risk
22.	Kaban^55^	High risk	Unclear risk	Unclear risk	Unclear risk	Low risk
23.	Kitajima^56^	Unclear risk	Unclear risk	High risk	Low risk	Low risk
24.	Lin^57^	Low risk	Low risk	Unclear risk	Low risk	Low risk
25.	Manzoni^58^	Low risk	Unclear risk	High risk	Low risk	Low risk
26.	Marti^59^	Low risk	Low risk	Low risk	Low risk	Low risk
27.	Mihatsch^60^	Low risk	Low risk	Low risk	Low risk	Low risk
28.	Millar^61^	Unclear risk	Unclear risk	Unclear risk	Low risk	Low risk
29.	Mohan^62^	Low risk	Unclear risk	Unclear risk	Low risk	Low risk
30.	Oncel^63^	Low risk	Low risk	Low risk	Low risk	Low risk
31.	Oshiro^64^	Low risk	Low risk	Low risk	Low risk	Low risk
32.	Patole^65^	Low risk	Low risk	Low risk	Low risk	Low risk
33.	Qiao^66^	Low risk	Low risk	Low risk	Low risk	Low risk
34.	Rehman^67^	Low risk	Unclear risk	High risk	Low risk	Unclear risk
35.	Reuman^68^	High risk	High risk	High risk	Low risk	Unclear risk
36.	Rojas^69^	Low risk	Low risk	Low risk	Low risk	Low risk
37.	Romeo^70^	Low risk	High risk	High risk	Low risk	High risk
38.	Rouge^71^	Low risk	Low risk	Low risk	Low risk	Low risk
39.	Roy^72^	Low risk	Low risk	Low risk	Low risk	Low risk
40.	Sadowska-Krawczenko^73^	Low risk	Low risk	Low risk	Low risk	Low risk
41.	Saewngtawesin^74^	Unclear risk	Unclear risk	High risk	Low risk	Low risk
42.	Sari^75^	Low risk	Low risk	Unclear risk	Low risk	Low risk
43.	Serec^76^	Low risk	Low risk	Unclear risk	Low risk	Low risk
44.	Shadkam^77^	Low risk	Unclear risk	Unclear risk	Low risk	Low risk
45.	Speckels^80^	Low risk	Low risk	Low risk	Low risk	Low risk
46.	Stratiki^81^	Low risk	Unclear risk	Unclear risk	Low risk	Low risk
47.	Strus^82^	Low risk	Low risk	Low risk	Low risk	Low risk
48.	Tewari^83^	Low risk	Low risk	Low risk	Low risk	Low risk
49.	Totsu^84^	Low risk	Unclear risk	Low risk	Low risk	Low risk
50.	Wejryd^85^	Low risk	Low risk	Low risk	Low risk	Low risk
51.	Xu^86^	Low risk	Low risk	Low risk	High risk	High risk

### Outcomes

The effects of the intervention were compared between the studies that used probiotics containing *B. infantis* versus those that used probiotics without *B. infantis*.

#### (1) NEC ≥Stage II

**Fig. 2:**
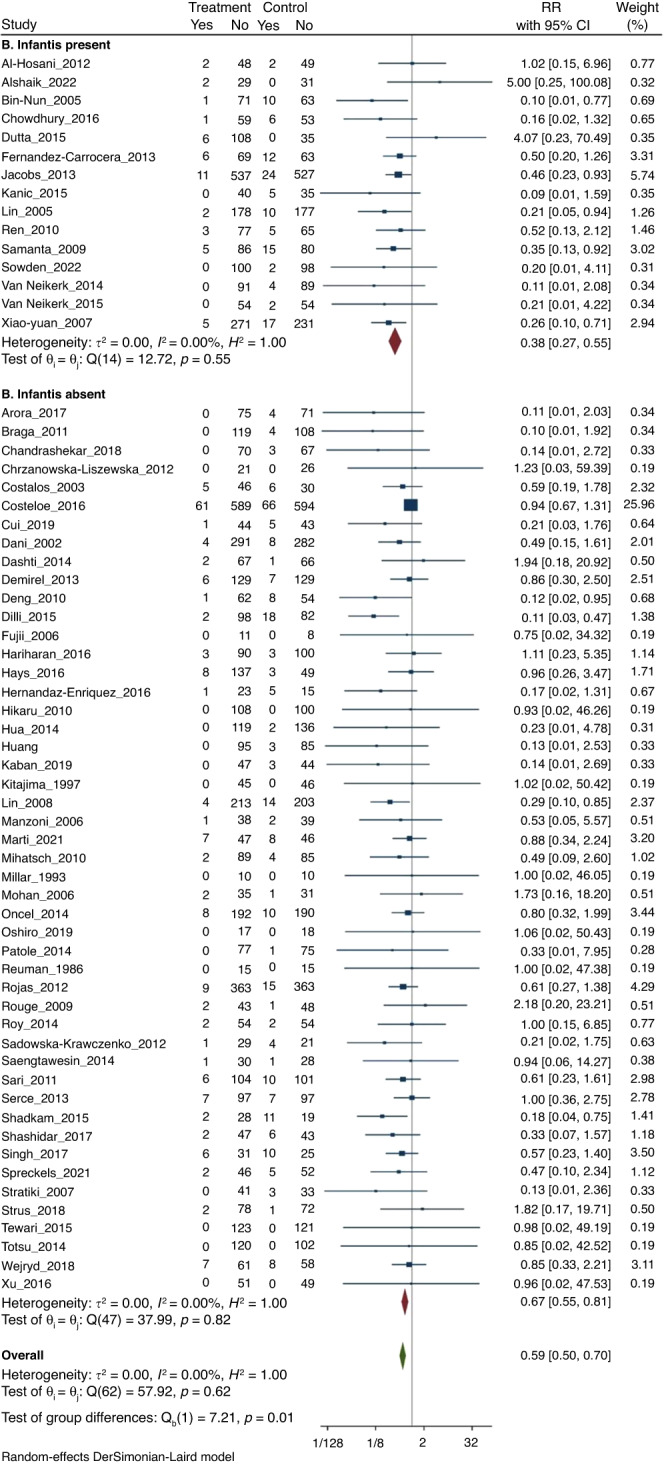
Forest plot. Probiotic supplementation to reduce NEC (≥Stage II).

Meta-analysis of all 63 RCTs found that probiotics decreased the risk of NEC (211/6394 [3.3%] vs 387/6170 [6.3%]); RR 0.59 (CI 0.50–0.70); *I*^2^ = 0% (Fig. 2).

On subgroup meta-analysis, 15 RCTs (*n* = 3626) that used probiotics containing *B. infantis (Subgroup A)* showed significant reduction in the incidence of NEC (44/1862 [2.4%] vs 114/1764 [6.5%]; RR 0.38; 95% CI, 0.27–0.55; *I*^2^ = 0% (Fig. 2). Subgroup meta-analysis of 48 RCTs (*n* = 8938) that used probiotics without *B. infantis* (subgroup B) also found significant reduction in the incidence of NEC (167/4532 [3.7%] vs 273/4406 [6.2%]; RR 0.67; 95% CI, 0.55–0.81; *I*^2^ = 0% (Fig. 2).

The p-value for subgroup differences was 0.01, which suggested that the beneficial effects are more pronounced in studies that had B*. infantis* as a component of the probiotic product.

To determine the publication bias, various statistical tests were used. Harbord (*p* = 0.203) and Begg’s (*p* = 0.577) tests showed no publication bias exists but the Egger’s test (p = 0.010) did. Further, trim & fill analysis imputed 9 potentially missing studies (Fig. 5a); however, the final results after including the imputed studies was still significant (RR = 0.60; 95% CI (0.49–0.73).

#### (2) LOS

**Fig. 3:**
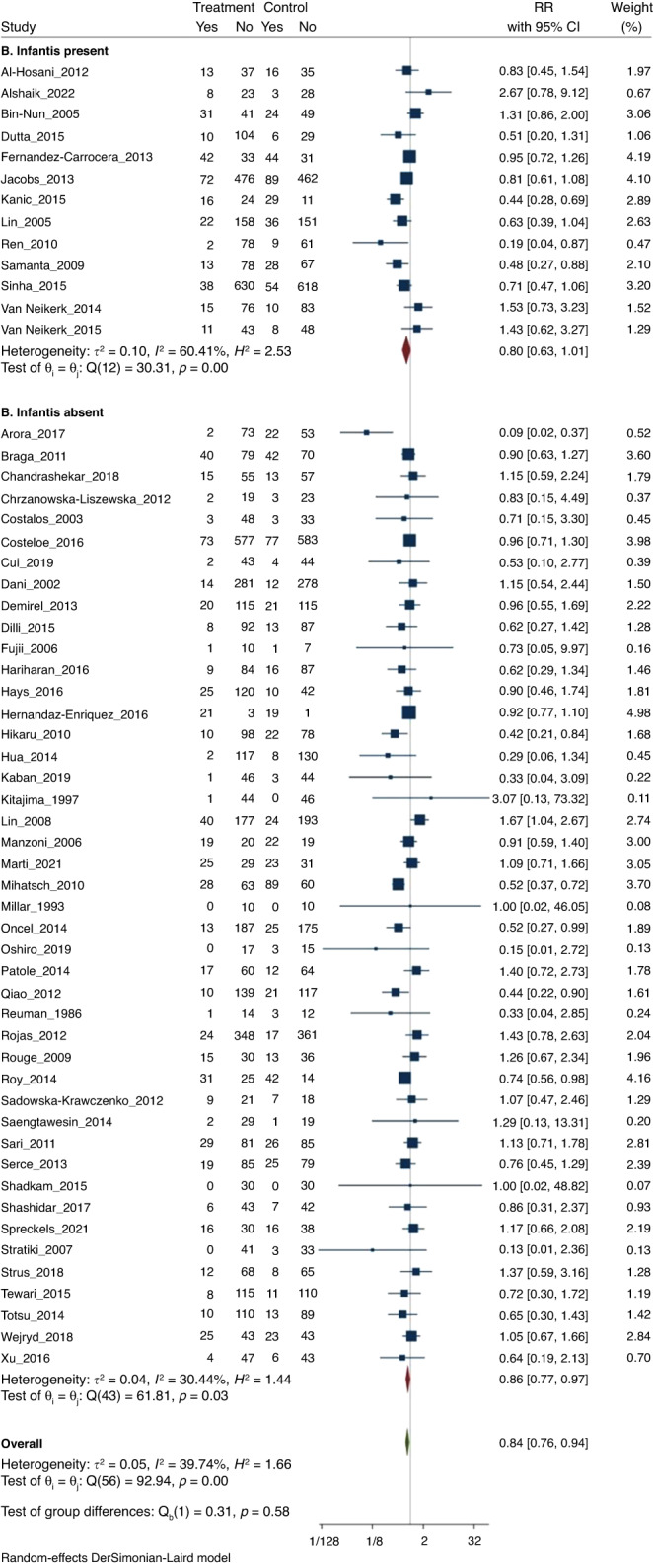
Forest plot. Probiotic supplementation to reduce LOS.

Meta-analysis of all 57 RCTs found that probiotics decreased the risk of LOS (905/6472 [14%] vs 1025/6277 [16.3%]); RR 0.84 (CI 0.76–0.94); *I*^2^ = 40% (Fig. 3).

On subgroup meta-analysis, 13 RCTs (*n* = 4123) that used probiotics containing *B. infantis* (subgroup A) showed that probiotics reduced the incidence of LOS (293/2094 [14%] vs 356/2029 [17.5%]; RR 0.80; 95% CI, 0.63–1.01; *I*^2^ = 60% (Fig. 3). Subgroup meta-analysis of 44 RCTs (*n* = 8626) that used probiotics without *B. infantis* (subgroup B) also found reduction of LOS (612/4378 [14%] vs 669/4248 [15.7%]; RR 0.86; 95% CI, 0.77–0.97; *I*^2^ = 30% (Fig. 3).

The *p* value for subgroup differences was 0.58, which suggested that the beneficial effects for the prevention of LOS were similar irrespective of whether B*. infantis* was a component of the probiotic product or not.

In the analysis for any publication bias. Harbord (*p* = 0.149) and Begg’s (*p* = 0.188) tests showed no publication bias exists but the Egger’s test (*p* = 0.088) did. Further, trim & fill analysis imputed 5 potentially missing studies (Fig. 5b); however, the final results after including the imputed studies was still significant [0.87; 95% CI (0.77–0.97).

#### (3) All-cause mortality

**Fig. 4:**
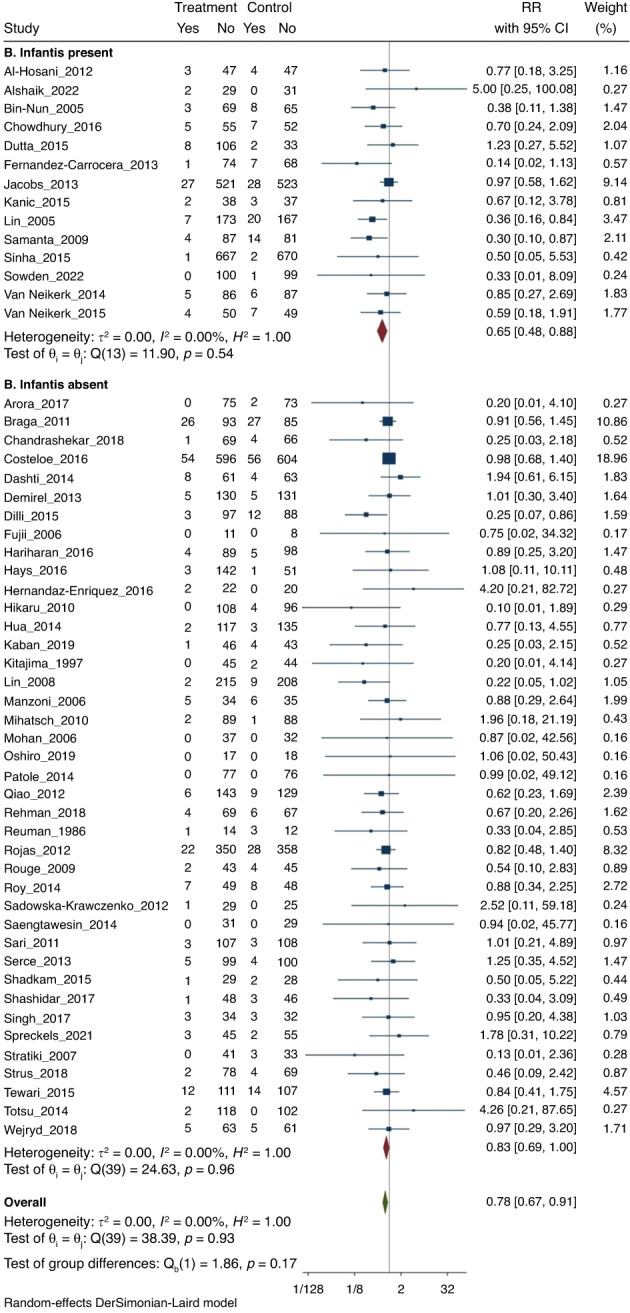
Forest plot. Probiotic supplementation to reduce mortality.

Meta-analysis of all 54 RCTs found that probiotics decreased the risk of all-cause mortality (270/6043 [4.5%] vs 355/5872 [6%]); RR 0.78 (CI 0.67–0.91); *I*^2^ = 0% (Fig. 4).

On subgroup meta-analysis, 14 RCTs (*n* = 4292) that used probiotics containing *B. infantis* (subgroup A) showed significant reduction of all-cause mortality (72/2174 [3.3%] vs 109/2118 [5.1%]; RR 0.65; 95% CI, 0.48–0.88; *I*^2^ = 0% (Fig. 4). Subgroup meta-analysis of 40 RCTs (*n* = 7623) that used probiotics without *B. infantis* (subgroup B) also found reduction of all-cause mortality (198/3869 [5.1%] vs 246/3754 [6.5%]; RR 0.83; 95% CI, 0.69–1.00; *I*^2^ = 0% (Fig. 4).

The *p* value for subgroup differences was 0.17, which suggested that the beneficial effects for the reduction in mortality were similar irrespective of whether B*. infantis* was a component of the probiotic product or not.

**Fig. 5:**
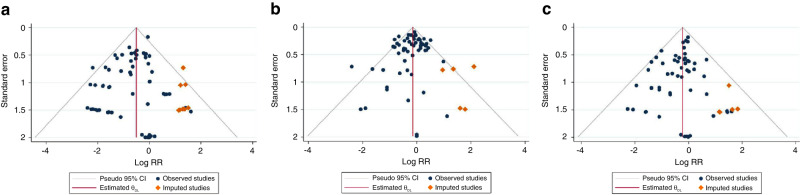
Trim and fill funnel plots for NEC, sepsis and mortality. **a** Trim and fill funnel plot for publication bias for NEC. **b** Trim and fill funnel plot for publication bias for sepsis. **c** Trim and fill funnel plot for publication bias for mortality.

In the analysis for any publication bias, Harbord (*p* = 0.151), Begg’s (*p* = 0.560) and Egger’s tests (*p* = 0.100) showed that there is no publication bias. However, the trim & fill analysis imputed 5 potentially missing studies. Meta-analysis after incorporating the results of imputed studies found results that were similar to the primary analysis [(RR = 0.80; 95% CI (0.69–0.93)] (Fig. 5c).

For studies in which *B. infantis* was a component of the probiotic supplement, the overall GRADE of evidence was high for the outcomes of mortality and NEC and moderate for LOS (Table 3a). For studies in which *B. infantis* was not a component of the probiotic supplement, the overall GRADE of evidence was high for all the outcomes of NEC, LOS, and mortality (Table 3b).

**Table 3:** (a) Summary of findings according to GRADE guidelines for RCTs that used *B. infantis*; (b) summary of findings according to GRADE guidelines for RCTs without *B. infantis*.

(a)											
Certainty assessment							No. of patients		Effect		Certainty
No. of studies	Study design	Risk of bias	Inconsistency	Indirectness	Imprecision	Other considerations	probiotics containing *B. infantis*	No probiotics	Relative (95% CI)	Absolute (95% CI)	
Necrotising enterocolitis											
15	Randomised trials	Serious^a^	Not serious	Not serious	Not serious	Strong association	44/1862 (2.4%)	114/1764 (6.5%)	RR 0.38 (0.27–0.55)	40 fewer per 1000 (from 47 fewer to 29 fewer)	⨁⨁⨁⨁ High
Late-onset sepsis											
13	Randomised trials	Serious^a^	Not serious^b^	Not serious	Not serious	None	293/2094 (14.0%)	356/2029 (17.5%)	RR 0.80 (0.63–1.01)	35 fewer per 1000 (from 65 fewer to 2 more)	⨁⨁⨁◯ Moderate
All-cause mortality											
14	Randomised trials	Serious^a^	Not serious	Not serious	Not serious	None	72/2174 (3.3%)	109/2118 (5.1%)	RR 0.65 (0.48–0.88)	18 fewer per 1000 (from 27 fewer to 6 fewer)	⨁⨁⨁◯ Moderate

(b)											
Certainty assessment							No. of patients		Effect		Certainty
No. of studies	Study design	Risk of bias	Inconsistency	Indirectness	Imprecision	Other considerations	probiotics without *B. infantis*	No probiotics	Relative (95% CI)	Absolute (95% CI)	
Necrotising enterocolitis											
48	Randomised trials	Serious^a^	Not serious	Not serious	Not serious	None	167/4532 (3.7%)	273/4406 (6.2%)	RR 0.67 (0.55–0.81)	20 fewer per 1000 (from 28 fewer to 12 fewer)	⨁⨁⨁◯ Moderate
Late-onset sepsis											
44	Randomised trials	Serious^a^	Not serious	Not serious	Not serious	None	612/4378 (14.0%)	669/4248 (15.7%)	RR 0.90 (0.81–1.00)	16 fewer per 1000 (from 30 fewer to 0 fewer)	⨁⨁⨁◯ Moderate
All cause mortality											
40	Randomised trials	Serious^a^	Not serious	Not serious	not serious	None	198/3869 (5.1%)	246/3754 (6.6%)	RR 0.83 (0.69–1.00)	11 fewer per 1000 (from 20 fewer to 0 fewer)	⨁⨁⨁◯ Moderate

*CI* confidence interval, *RR* risk ratio.

^a^Publication bias.

^b^*I*^2^ statistic 40%.

## Discussion

Our systematic review that included 67 RCTs (*n* = 14,606) found that probiotic supplementation significantly reduced the risk of NEC≥ Stage II, LOS and all-cause mortality in preterm infants. Specific to our aim, the sub-group meta-analysis of RCTs that used probiotics containing *B. infantis* showed even more favourable results, especially for the prevention of NEC (≥Stage II). These results provide indirect evidence that probiotics that include *B. infantis* may be more beneficial in preterm infants than those not including *B. infantis*.

Our results are supported by a recent non-randomised study by Tobias et al, involving 483 VLBW infants. Supplementation of *B. infantis* was associated with a significant reduction in NEC (≥Stage II) and NEC-related mortality.^87^ The *B. infantis* cohort had a 73% reduction of NEC (≥ stage II) compared with the no *B. infantis* cohort (adjusted RR, 0.27; 95% CI, 0.094–0.614; *p* < 0.01).

A prospective study by Nguyen et al. has evaluated the effect of *B. infantis* administration on gut microbiota, nosocomial acquired antibiotic resistance and enteric inflammation in preterm infants with gestation <32 weeks and/or birth weight <1500 g.^88^ Infants supplemented with *B. infantis* had lower enteric inflammation after adjusting for other clinical variables in multivariate modelling. In contrast, Kochjancic et al. reported that probiotic (*B. infantis* and *Lactobacillus acidophilus*) supplementation did not reduce the risk of NEC in neonates with duct-dependent congenital heart disease (CHD).^89^ The lack of benefits of probiotics may relate to the small sample size (*n* = 15) and retrospective design of the study, lack of concurrence of NEC and duct-dependent CHD, and the fact that the majority of infants were born at term.

Discussing the physiological characteristics of *B. infantis* is important as probiotic effects are species and strain- specific. Ward et al. and LoCascio et al. reported that given their complex structure, the HMOs cannot be metabolised by the infant or most of the bacteria in the infant’s gut, as they lack the necessary enzymes for the purpose.^90,91^ *B. infantis* has been shown to grow in vitro using HMO as the sole carbon source, reaching a cell density 3-fold higher than *B. longum* subsp. longum, *B. breve*, *B. bifidum*, and *B. adolescentis*. Low pH is a critical factor in preventing the invasion and overgrowth of harmful bacteria in the infant gut, a process known as colonisation resistance. Henrick et al. reported that *B. infantis* supplementation significantly lowered faecal pH in breastfed infants compared to controls.^92^ Underwood et al. reported that HMO metabolism by *B. infantis* produces short-chain fatty acids (SCFA), such as acetate, which play an important role in nutrition and intestinal and immune development, facilitate direct binding to intestinal cells, and stimulate anti-inflammatory/inhibits pro-inflammatory cytokine release by intestinal cells.^93^ Meng et al. reported that *B. infantis* contributes to maintaining of gut barrier integrity through indole 3-lactic acid (ILA), a metabolite of tryptophan, and may protect gut epithelium by activating the aryl hydrogen receptor, which can further promote intestinal immune function.^10^ In addition to their role in the gut, SCFAs produced by *B. infantis* can enter circulation and directly affect the adipose tissue, lungs, brain, and liver, inducing overall beneficial metabolic effects.^94^ Animal studies by Bergmann et al. suggested that *B. infantis* can potentially protect against excessive intestinal inflammation which is implicated in the pathogenesis of NEC in preterm infants.^95^ Given that probiotics are live organisms, a major concern is the risk of sepsis due to the administered probiotic organism. Although there are few case reports of bacteraemia caused by the Bifidobacteria,^96,97^ it is reassuring to note that none of the RCTs included in our review that used probiotics reported probiotic related sepsis. However, current evidence is limited for estimating the risk of probiotic sepsis.^98,99^ In 2007, the European Food Safety Authority (EFSA) assigned qualified presumption of safety (QPS) status to the bacterial species *B. longum*, which includes subspecies *infantis*, indicating that this taxonomic group does not carry safety concerns.^100^ The QPS status, which applies to all strains of *B. infantis* indicates that none of these has been associated with human clinical disease. However, it should not lead to complacency, and constant microbiological surveillance is essential to identify and treat sepsis that may occur due to the administered probiotic organism.

To our knowledge, ours is the first systematic review related to *B. infantis* in preterm infants. Our results will help in guiding research for using *B. infantis* as a probiotic in preterm infants. This is also one of the largest systematic reviews of probiotics in preterm infants involving 14,606 preterm infants, which is close to the recent network meta-analysis (3) and 3800 infants more than the Cochrane review.^101^

Our systematic review has some limitations. Since only 16 RCTs used probiotics containing *B. infantis* and the remaining 51 RCTs used a probiotic that did not contain *B. infantis*, it resulted in a large discrepancy between the number of participants between these two groups. Furthermore, none of the included RCTs used *B. infantis* as the sole probiotic. Instead, they used a mixture of probiotic organisms with variable doses of *B. infantis*. Thus, the observed benefits cannot be attributed definitively to *B. infantis*.

In conclusion, our systematic review of RCTs provides indirect evidence that the beneficial effects for the prevention of NEC are more pronounced if *B. infantis* is a component of the probiotic product compared to studies in which *B. infantis* is not a component. However, given the limitations to the evidence, adequately powered RCTs are necessary to confirm the benefits and safety of *B. infantis* in preterm infants. Such RCTs could compare (1) *B. infantis* versus Placebo or (2) *B. infantis* as a component of a multi-strain probiotic product versus the same multi-strain probiotic but without *B. infantis*.

### Supplementary information

PRISMA_checklist

## Data Availability

Data sharing not applicable to this article as no datasets were generated or analysed during the current study.

## References

[1] Lee CC (2021). Gut dysbiosis, bacterial colonization and translocation, and neonatal sepsis in very-low-birth-weight preterm infants. Front. Microbiol..

[2] Pineiro M (2008). FAO Technical meeting on prebiotics. J. Clin. Gastroenterol..

[3] Morgan RL, Preidis GA, Kashyap PC, Weizman AV, Sadeghirad B (2020). McMaster Probiotic, Prebiotic, and Synbiotic Work Group. Probiotics reduce mortality and morbidity in preterm, low-birth-weight infants: a systematic review and network meta-analysis of randomized trials. Gastroenterology.

[4] Underwood MA (2019). Probiotics and the prevention of necrotizing enterocolitis. J. Pediatr. Surg. Mar..

[5] McFarland LV, Evans CT, Goldstein EJC (2018). Strain-specificity and disease-specificity of probiotic efficacy: a systematic review and meta-analysis. Front. Med..

[6] Chichlowski M, Shah N, Wampler JL, Wu SS, Vanderhoof JA (2020). *Bifidobacterium longum* Subspecies *infantis* (*B. infantis*) in pediatric nutrition: current state of knowledge. Nutrients.

[7] Underwood MA, German JB, Lebrilla CB, Mills DA (2015). *Bifidobacterium longum* subspecies *infantis*: champion colonizer of the infant gut. Pediatr. Res..

[8] Frese SA (2017). Persistence of supplemented *Bifidobacterium longum* subsp. *Infantis* EVC001 in breastfed infants. mSphere.

[9] Chichlowski M (2012). Bifidobacteria isolated from infants and cultured on human milk oligosaccharides affect intestinal epithelial function. J. Pediatr. Gastroenterol. Nutr..

[10] Meng D (2020). Indole-3-lactic acid, a metabolite of tryptophan, secreted by *Bifidobacterium longum* subspecies *infantis* is anti-inflammatory in the immature intestine. Pediatr. Res..

[11] Athalye-Jape G (2022). Effect of single versus multistrain probiotic in extremely preterm infants: a randomised trial. BMJ Open Gastroenterol..

[12] Higgins, J. P. et al. *Cochrane Handbook for Systematic Reviews of Interventions Version 6.3 (Updated 2022)* (Cochrane, 2022).

[13] Moher D, Altman DG, Liberati A, Tetzlaff J (2011). PRISMA statement. Epidemiology.

[14] Walsh M, Kliegman R (1986). Necrotizing enterocolitis: treatment based on staging criteria. Pediatr. Clin. North Am..

[15] Egger M, Davey Smith G, Schneider M, Minder C (1997). Bias in meta-analysis detected by a simple, graphical test. BMJ.

[16] Harbord RM, Egger M, Sterne JA (2006). A modified test for small-study effects in meta-analyses of controlled trials with binary endpoints. Stat. Med..

[17] Begg CB, Mazumdar M (1994). Operating characteristics of a rank correlation test for publication bias. Biometrics.

[18] Shi L, Lin L (2019). The trim-and-fill method for publication bias: practical guidelines and recommendations based on a large database of meta-analyses. Medicine.

[19] Schünemann, H., Brozek, J., Guyatt, G. & Oxman, A. (eds) *Handbook for Grading the Quality of Evidence and the Strength of Recommendations using the GRADE Approach (Updated October 2013)* (GRADE Working Group, 2013).

[20] Al-Hosni M (2012). Probiotics-supplemented feeding in extremely low-birth-weight infants. J. Perinatol..

[21] Alshaikh, B. et al. Multi-strain probiotics for extremely preterm infants: a randomized controlled trial. *Pediatr. Res*. **92**, 1663–1670 (2022). 10.1038/s41390-022-02004-z 35314794

[22] Bin-Nun A (2005). Oral probiotics prevent necrotizing enterocolitis in very low birth weight neonates. J. Pediatr..

[23] Chowdhury T (2016). Efficacy of probiotics versus placebo in the prevention of necrotizing enterocolitis in preterm very low birth weight infants: a double-blind randomized controlled trial. J. Coll. Physicians Surg. Pak..

[24] Dutta S, Ray P, Narang A (2015). Comparison of stool colonization in premature infants by three dose regimes of a probiotic combination: a randomized controlled trial. Am. J. Perinatol..

[25] Fernández-Carrocera LA (2013). Double-blind, randomised clinical assay to evaluate the efficacy of probiotics in preterm newborns weighing less than 1500 g in the prevention of necrotising enterocolitis. Arch. Dis. Child. Fetal Neonatal Ed..

[26] Jacobs SE (2013). ProPrems Study Group. Probiotic effects on late-onset sepsis in very preterm infants: a randomized controlled trial. Pediatrics.

[27] Kanic Z, Micetic Turk D, Burja S, Kanic V, Dinevski D (2015). Influence of a combination of probiotics on bacterial infections in very low birthweight newborns. Wien. Klin. Wochenschr..

[28] Lin HC (2005). Oral probiotics reduce the incidence and severity of necrotizing enterocolitis in very low birth weight infants. Pediatrics.

[29] Ren YF, Wang LL (2010). Effects of probiotics on intestinal bacterial colonization in premature infants. Zhongguo Dang Dai Er Ke Za Zhi.

[30] Samanta M (2009). Prophylactic probiotics for prevention of necrotizing enterocolitis in very low birth weight newborns. J. Trop. Pediatr..

[31] Sinha A (2015). Role of probiotics VSL#3 in prevention of suspected sepsis in low birthweight infants in India: a randomised controlled trial. BMJ Open.

[32] Sowden M, van Niekerk E, Bulabula ANH, Twisk J, van Weissenbruch MM (2022). Effect of a multi-strain probiotic on growth and time to reach full feeds in preterm neonates. Nutrients.

[33] Van Niekerk E, Kirsten GF, Nel DG, Blaauw R (2014). Probiotics, feeding tolerance, and growth: a comparison between HIV-exposed and unexposed very low birth weight infants. Nutrition.

[34] Van Niekerk E, Nel DG, Blaauw R, Kirsten GF (2015). Probiotics reduce necrotizing enterocolitis severity in HIV-exposed premature infants. J. Trop. Pediatr..

[35] Xiao-yuan Z, Lian-qiao LI, Xuan-xuan GAO, Li-duan SU (2007). Relative factors of neonatal necrotizing enterocolitis and preventive effect of microeco-preparation. J. Appl. Clin. Pediatr..

[36] Arora S, Khurana MS, Saini R (2017). To study the role of probiotics in the prevention of necrotizing enterocolitis in preterm neonates. Int. J. Contemp. Pediatr..

[37] Braga TD, Da Silva GAP, De Lira PIC, De Carvalho Lima M (2011). Efficacy of *Bifidobacterium breve* and *Lactobacillus casei* oral supplementation on necrotizing enterocolitis in very-low-birth-weight preterm infants: a double-blind, randomized, controlled trial. Am. J. Clin. Nutr..

[38] Chandrashekar GS, Shettigar S, Varghese TC (2018). Role of probiotics in prevention of necrotizing enterocolitis in preterm neonates. Indian J. Child Health.

[39] Chrzanowska-Liszewska D, Seliga-Siwecka J, Kornacka MK (2012). The effect of *Lactobacillus rhamnosus* GG supplemented enteral feeding on the microbiotic flora of preterm infants-double blinded randomized control trial. Early Hum. Dev..

[40] Costalos C (2003). Enteral feeding of premature infants with *Saccharomyces boulardii*. Early Hum. Dev. Nov..

[41] Costeloe K, Hardy P, Juszczak E, Wilks M, Millar MR (2015). *Bifidobacterium breve* BBG-001 in very preterm infants:a randomised controlled phase 3 trial. Lancet.

[42] Cui X, Shi Y, Gao S, Xue X, Fu J (2019). Effects of *Lactobacillus reuteri* DSM 17938 in preterm infants: a double-blinded randomized controlled study. Ital. J. Pediatr..

[43] Dani C, Biadaioli R, Bertini G, Martelli E, Rubaltelli FF (2002). Probiotics feeding in prevention of urinary tract infection, bacterial sepsis and necrotizing enterocolitis in preterm infants. A prospective double-blind study. Biol. Neonate.

[44] Dashti AS (2014). Prophylactic probiotics for prevention of necrotizing enterocolitis (NEC) in low birth weight neonates. Arch. Pediatr. Infect. Dis..

[45] Demirel G, Erdeve O, Celik IH, Dilmen U (2013). *Saccharomyces boulardii* for prevention of necrotizing enterocolitis in preterm infants: a randomized, controlled study. Acta Paediatr..

[46] Deng J, Chen K (2010). Early minimal feeding combined with probiotics to prevent necrotizing enterocolitis in preterm infant. Chin. J. Mod. Drug Appl..

[47] Dilli D (2015). The ProPre-Save Study: effects of probiotics and prebiotics alone or combined on necrotizing enterocolitis in very low birth weight infants. J. Pediatr. Mar..

[48] Fujii T (2006). Bifidobacterium breve enhances transforming growth factor beta1 signaling by regulating Smad7 expression in preterm infants. J. Pediatr. Gastroenterol. Nutr..

[49] Hariharan D, Balasubramanian L, Kannappan V, Veluswami G (2016). Probiotic supplementation in VLBW preterm infants improves feeding tolerance and reduces risk of Gram negative sepsis. J. Pediatr. Gastroenterol. Nutr..

[50] Hays S (2016). Probiotics and growth in preterm infants: a randomized controlled trial, PREMAPRO study. Clin. Nutr..

[51] Hernandez-Enriquez NP, Rosas-Sumano AB, Monzoy-Ventre MA, Galicia-Flores L (2016). *Lactobacillus reuteri* DSM 17938 in preventing necrotizing enterocolitis in preterm newborns. Pilot study of efficacy and safety. Rev. Mex. Pediatr..

[52] Hikaru U (2010). Bifidobacteria prevents preterm infants from developing infection and sepsis. Int. J. Probiotics Prebiotics.

[53] Hua XT, Tang J, Mu DZ (2014). Effect of oral administration of probiotics on intestinal colonization with drug-resistant bacteria in preterm infants. Chin. J. Contemp. Pediatr..

[54] Huang B, Yang H, Huang X (2009). Probiotics supplementation for prevention of necrotizing enterocolitis in very low-birth-weight neonates: a randomized, controlled trial. J. Guangdong Med. Coll..

[55] Kaban RK (2019). *Lactobacillus reuteri* DSM 17938 improves feeding intolerance in preterm infants. Pediatr. Gastroenterol. Hepatol. Nutr..

[56] Kitajima H (1997). Early administration of *Bifidobacterium breve* to preterm infants: randomised controlled trial. Arch. Dis. Child. Fetal Neonatal Ed..

[57] Lin HC (2008). Oral probiotics prevent necrotizing enterocolitis in very low birth weight preterm infants: a multicenter, randomized, controlled trial. Pediatrics.

[58] Manzoni P (2006). Oral supplementation with *Lactobacillus casei* subspecies rhamnosus prevents enteric colonization by Candida species in preterm neonates: a randomized study. Clin. Infect. Dis..

[59] Martí M (2021). Effects of *Lactobacillus reuteri* supplementation on the gut microbiota in extremely preterm infants in a randomized placebo-controlled trial. Cell Rep. Med..

[60] Mihatsch WA, Vossbeck S, Eikmanns B, Hoegel J, Pohlandt F (2010). Effect of Bifidobacterium lactis on the incidence of nosocomial infections in very-low-birth-weight infants: a randomized controlled trial. Neonatology.

[61] Millar MR, Bacon C, Smith SL, Walker V, Hall MA (1993). Enteral feeding of premature infants with Lactobacillus GG. Arch. Dis. Child..

[62] Mohan R (2006). Effects of *Bifidobacterium lactis* Bb12 supplementation on intestinal microbiota of preterm infants: a double-blind, placebo-controlled, randomized study. J. Clin. Microbiol.

[63] Oncel MY (2014). *Lactobacillus reuteri* for the prevention of necrotising enterocolitis in very low birthweight infants: a randomised controlled trial. Arch. Dis. Child. Fetal Neonatal Ed..

[64] Oshiro T (2019). Bifidobacterium supplementation of colostrum and breast milk enhances weight gain and metabolic responses associated with microbiota establishment in very-preterm infants. Biomed. Hub..

[65] Patole, S. et al. Effect of *Bifidobacterium breve* M-16V supplementation on fecal bifidobacteria in preterm neonates-a randomised double blind placebo controlled trial. *PLoS ONE* **9**, e89511 (2014). 10.1371/journal.pone.0089511 PMC3940439 24594833

[66] Qiao LX, Zhu WY, Zhang HY, Wang H (2017). Effect of early administration of probiotics on gut microflora and feeding in pre-term infants: a randomized controlled trial. J. Matern. Fetal Neonatal Med..

[67] Rehman SU, Iqbal A, Ali W (2018). Role of probiotics in reducing frequency of necrotizing enterocolitis in preterm neonates. Pak. Pediatr. J..

[68] Reuman PD (1986). Lack of effect of Lactobacillus on gastrointestinal bacterial colonization in premature infants. Pediatr. Infect. Dis..

[69] Rojas MA (2012). Prophylactic probiotics to prevent death and nosocomial infection in preterm infants. Pediatrics.

[70] Romeo MG (2011). Role of probiotics in the prevention of the enteric colonization by Candida in preterm newborns: incidence of late-onset sepsis and neurological outcome. J. Perinatol..

[71] Rougé C (2009). Oral supplementation with probiotics in very-low-birth-weight preterm infants: a randomized, double-blind, placebo-controlled trial. Am. J. Clin. Nutr..

[72] Roy A, Chaudhuri J, Sarkar D, Ghosh P, Chakraborty S (2014). Role of enteric supplementation of probiotics on late-onset sepsis by Candida species in preterm low birth weight neonates: a randomized, double blind, placebo-controlled trial. North Am. J. Med. Sci..

[73] Sadowska-Krawczenko I, Korbal P, Polak A, Wietlicka-Piszcz M, Szajewska H (2012). *Lactobacilllus rhamnosus* ATC A07FA for preventing necrotizing enterocolitis in very-low-birth-weight preterm infants: a randomized controlled trial (preliminary results) [Polish]. Pediatr. Pol..

[74] Saengtawesin V, Tangpolkaiwalsak R, Kanjanapattankul W (2014). Effect of oral probiotics supplementation in the prevention of necrotizing enterocolitis among very low birth weight preterm infants. J. Med. Assoc. Thail..

[75] Sari FN (2011). *Lactobacillus sporogenes* for prevention of necrotizing enterocolitis in very low-birth weight infants: a randomized, controlled trial. Eur. J. Clin. Nutr..

[76] Serce O, Benzer D, Gursoy T, Karatekin G, Ovali F (2013). Efficacy of *Saccharomyces boulardii* on necrotizing enterocolitis or sepsis in very low birth weight infants: a randomised controlled trial. Early Hum. Dev..

[77] Shadkam MN, Jalalizadeh F, Nasiriani K (2015). Effects of probiotic *Lactobacillus reuteri* (DSM 17938) on the incidence of necrotizing enterocolitis in very low birth weight premature infants. Iran. J. Neonatol..

[78] Shashidhar A, Suman Rao PN, Nesargi S, Bhat S, Chandrakala BS (2017). Probiotics for promoting feed tolerance in very low birth weight neonates - a randomized controlled trial. Indian Pediatr..

[79] Dongol Singh SS, Klobassa DS, Resch B, Urlesberger B, Shrestha RP (2017). Placebo controlled introduction of prophylactic supplementation of probiotics to decrease the incidence of necrotizing enterocolitis at Dhulikhel Hospital in Nepal. Kathmandu Univ. Med. J..

[80] Spreckels JE (2021). *Lactobacillus reuteri* colonisation of extremely preterm infants in a randomised placebo-controlled trial. Microorganisms.

[81] Stratiki Z (2007). The effect of a Bifidobacter supplemented bovine milk on intestinal permeability of preterm infants. Early Hum. Dev..

[82] Strus M (2018). Effects of oral probiotic supplementation on gut Lactobacillus and Bifidobacterium populations and the clinical status of low-birth-weight preterm neonates: a multicenter randomized, double-blind, placebo-controlled trial. Infect. Drug Resist..

[83] Tewari VV, Dubey SK, Gupta G (2015). *Bacillus clausii* for prevention of late-onset sepsis in preterm infants: a randomized controlled trial. J. Trop. Pediatr..

[84] Totsu S, Terahara M, Kusuda S (2018). Probiotics and the development of very low birthweight infants: follow-up study of a randomised trial. BMJ Paediatr. Open.

[85] Wejryd E, Marchini G, Frimmel V, Jonsson B, Abrahamsson T (2019). Probiotics promoted head growth in extremely low birthweight infants in a double-blind placebo-controlled trial. Acta Paediatr..

[86] Xu L (2016). A double-blinded randomized trial on growth and feeding tolerance with *Saccharomyces boulardii* CNCM I-745 in formula-fed preterm infants. J. Pediatr..

[87] Tobias J (2022). *Bifidobacteriumlongum* subsp. *infantis* EVC001 administration is associated with a significant reduction in the incidence of necrotizing enterocolitis in very low birth weight infants. J. Pediatr..

[88] Nguyen M (2021). Impact of probiotic *B. infantis* EVC001 feeding in premature infants on the gut microbiome, nosocomially acquired antibiotic resistance, and enteric inflammation. Front. Pediatr..

[89] Kocjancic L, Bührer C, Berger F, Boos V (2020). Effect of a dual-strain probiotic on necrotizing enterocolitis in neonates with ductal-dependent congenital heart disease: a retrospective cohort study. Neonatology.

[90] Ward RE, Ninonuevo M, Mills DA, Lebrilla CB, German JB (2007). In vitro fermentability of human milk oligosaccharides by several strains of bifidobacteria. Mol. Nutr. Food Res..

[91] LoCascio RG (2009). A versatile and scalable strategy for glycoprofiling bifidobacterial consumption of human milk oligosaccharides. Microb. Biotechnol..

[92] Henrick BM (2018). Elevated fecal pH indicates a profound change in the breastfed infant gut microbiome due to reduction of Bifidobacterium over the past century. mSphere.

[93] Underwood MA (2014). *Bifidobacterium longum* subsp. *infantis* in experimental necrotizing enterocolitis: alterations in inflammation, innate immune response, and the microbiota. Pediatr. Res..

[94] Koh A, De Vadder F, Kovatcheva-Datchary P, Bäckhed F (2016). From dietary fiber to host physiology: short-chain fatty acids as key bacterial metabolites. Cell.

[95] Bergmann KR (2013). Bifidobacteria stabilize claudins at tight junctions and prevent intestinal barrier dysfunction in mouse necrotizing enterocolitis. Am. J. Pathol..

[96] Esaiassen E (2016). *Bifidobacterium longum* subspecies *infantis* bacteremia in 3 extremely preterm infants receiving probiotics. Emerg. Infect. Dis..

[97] Bertelli C (2015). *Bifidobacterium longum* bacteremia in preterm infants receiving probiotics. Clin. Infect. Dis..

[98] Underwood MA (2022). *Bifidobacterium infantis*, necrotizing enterocolitis, death, and the role of parents in the NICU. J. Pediatr..

[99] Underwood MA, Umberger E, Patel RM (2020). Safety and efficacy of probiotic administration to preterm infants: ten common questions. Pediatr. Res..

[100] EFSA. (2007). Introduction of a Qualified Presumption of Safety (QPS) approach for assessment of selected microorganisms referred to EFSA—Opinion of the Scientific Committee. EFSA J..

[101] Sharif S, Meader N, Oddie SJ, Rojas-Reyes MX, McGuire W (2020). Probiotics to prevent necrotising enterocolitis in very preterm or very low birth weight infants. Cochrane Database Syst. Rev..

